# Virulence and antimicrobial resistance factors in *Salmonella enterica* serotypes isolated from pigs and chickens in central Chile

**DOI:** 10.3389/fvets.2022.971246

**Published:** 2022-09-20

**Authors:** Patricio Retamal, Joaquim Gaspar, María Belén Benavides, Leonardo Saenz, Nicolás Galarce, Trinidad Aravena, Javiera Cornejo, Lisette Lapierre

**Affiliations:** ^1^Departamento de Medicina Preventiva Animal, Facultad de Ciencias Veterinarias y Pecuarias, Universidad de Chile, Santiago, Chile; ^2^Magister en Ciencias Animales y Veterinarias, Facultad de Ciencias Veterinarias y Pecuarias, Universidad de Chile, Santiago, Chile; ^3^Departamento de Ciencias Biológicas, Facultad de Ciencias Veterinarias y Pecuarias, Universidad de Chile, Santiago, Chile; ^4^Escuela de Medicina Veterinaria, Facultad de Ciencias de la Vida, Universidad Andrés Bello, Santiago, Chile

**Keywords:** *Salmonella*, virulence, antimicrobial resistance, pigs, chicken, Chile

## Abstract

*Salmonella enterica* is a food-borne pathogen with a wide host-range that during decades has been of public health concern in developed and developing countries. In Chile, the poultry and pig industries represent the biggest contribution of meat consumption in the population, and sanitary regulations have been imposed for *Salmonella* control. The aim of this work was to determine and characterize *Salmonella* strains isolated from pigs and chicken raised on commercials farms in Chile. For this, isolates belonging to pigs (*n* = 46) and poultry (*n* = 57) were genotyped by two multiplex PCR reactions and virulotyped by the PCR detection of virulence-associated genes. In addition, isolates were serotyped and analyzed by the Kirby Bauer assay to determine their antimicrobial resistance phenotypes. From these analyses 52 genotypes, six serotypes and several multidrug resistance phenotypes and different combinations of virulence-associated genes were detected. These results suggest that *S. enterica* in pigs and poultry in central Chile should be monitored due to potential consequences in public and animal health.

## Introduction

*Salmonella enterica* is a foodborne pathogen that can cause enterocolitis with diarrhea, fever and abdominal pain in humans, as major clinical manifestations (1). Typically, *S. enterica* causes a self-limited digestive disease, although extra-intestinal infections of medical importance have also been reported, especially in patients of the high-risk group (2–4). Thus, the severity of human salmonellosis varies according to the serotype involved and the health status of the host (4, 5).

The *S. enterica* subsp. *enterica* has multiple reservoirs in domestic animals and wildlife, and its transmission to humans is usually by consumption of contaminated foodstuff, such as eggs or meat (6, 7). Colonized chickens and pigs, as well as contaminated products derived from those animals, are major sources of human salmonellosis in most countries. Thus, it is important to investigate and survey the epidemiology of this pathogen in those animal species for public health and poultry and pig husbandry (8). In a high proportion of cases, the meat is contaminated with feces during slaughter or dressing line, which constitutes the main risk factor for *Salmonella* to enter into the food chain (9). However, the food chain is not the only transmission pathway, being the direct animal-human contact a route that has been increasingly reported worldwide (10–12).

The expression of several virulence genes during the host-pathogen interaction allows the activation of adhesion and invasion mechanisms which determines the course of infection. These factors include flagella, capsule, plasmids, adhesins, and type 3 secretion systems (T3SS), among others. These virulence factors could enhance the adaptive capacity of some serotypes, representing *Salmonella* pathogenic mechanisms to infect, survive and establish the infection in the host (13, 14).

An additional factor of current concern is the emergence of antimicrobial resistance (AMR) in different *S. enterica* serotypes. In general, self-limited salmonellosis does not require antibiotic treatment; however, in patients with the invasive disease, it may be necessary to use antibiotics, especially cephalosporins and fluoroquinolones. Thus, AMR represent a risk for patients and for public health because therapies are less effective and the genes encoding resistance are positively selected and disseminated through mobilizable elements between bacterial strains of self and different species and sources (15, 16).

There are several zoonotic *Salmonella* serotypes that have a wide host range and cause infection and disease in humans, wildlife and domestic animals (4, 5). Thus, reporting the *Salmonella* serotypes involved in an outbreak turns essential for conducting an outbreak investigation and epidemiological surveillance (17). The traditional method of serotyping *Salmonella* isolates follow the Kauffmann-White scheme, based on the typification by serological discrimination, through the agglutination of somatic (O) and flagellar (H) antigens, and the capsular Vi antigen; describing more than 2,650 serotypes with diverse epidemiology and geographic distribution (18, 19). Depending on the serotype, this method could be slow, laborious and costly, which justifies the study of other faster and cost-effective *S. enterica* typing methodologies, such as the multiplex PCR (20, 21).

In South America, there is limited surveillance data on non-typhoidal *Salmonella* strains in food producing animals. Therefore, the objective of this study was to identify *S. enterica* serotypes in pigs and chickens from commercials farms located in central Chile and to characterize their AMR phenotypes, serotypes and virulotypes in order to provide relevant data for surveillance programs at the national and international level.

## Materials and methods

### Sample collection

In Chile, the national industrialized production of poultry and swine is concentrated in the central zone, specifically in three regions: Metropolitan Region, Libertador Bernardo O'Higgins Region and Maule Region, concentrating 79% of the national production (22, 23). A total of 500 stool samples from five pig farms and 300 stool samples from six poultry farms were individually collected during 2017 in central Chile. Animals in the final stage of production just before slaughter were sampled, by using sterile swabs with Cary Blair transport medium (Copan Diagnostics, Murrieta, CA, USA). Sampling was performed by personnel working on the same farms during a period of 12 months. After collection, all samples (*n* = 800) were immediately refrigerated and transported to the laboratory.

### Sample processing

In order to isolate *Salmonella* strains, samples were processed as previously reported (24). Briefly, swabs were placed into 5 mL of buffered peptone water (BPW, Beckton Dickinson, Franklin Lakes, NJ, USA) supplemented with 20 μg/mL of novobiocin (Sigma, St. Louis, MO, USA), and incubated for 24 h at 37°C. Then, 100 μL of the suspension were inoculated into modified semisolid Rappaport Vassiliadis basal medium (Oxoid, Basingstoke, UK) plates supplemented with 20 μg/mL of novobiocin and incubated for 24 or 48 h at 41°C. Cultures with bacterial growth were replated into xylose lysine deoxycholate agar (XLD, Beckton Dickinson, Franklin Lakes, NJ, USA) plates and incubated for 24 h at 37°C. Two suspicious colonies were initially subjected to traditional morphological and biochemical testing including Gram staining, and the use of triple sugar iron agar slopes and API 20E strips (bioMérieux, Marcy l'Etoile, France). Subsequently, if both colonies were *Salmonella*, only one of them was randomly selected for further analysis. From one colony, the genomic DNA was extracted using a commercial kit (Thermo Scientific, Waltham, MA, USA), following manufacturer's instructions. Concentration and quality (260/280 absorbance ratio) of the obtained extracted DNA was measured in a NANO-400 micro-spectrophotometer (Hangzhou Allsheng Instruments Co., Hangzhou, China). Samples with an absorbance ratio closest to the optimal range (1.8–2.0) (25) were kept at −20°C for further analyses.

### Molecular typing of *S. enterica* serovars

All identified *Salmonella* strains were subjected to a multiplex PCR protocol in order to identify genotypes (17, 20, 21). Each combination of amplified sequences was considered a genotype. Briefly, the method consists of two five-plex PCR reactions and one two-plex PCR reaction. The primers for each of the reactions and accession numbers for each of the gene are listed in Supplementary Table S1. The amplified regions of the genome mostly correspond to prophages and fimbrial clusters that are remarkably variable between serovars. These regions are potentially of great interest as they represent gene clusters that may have a serovar-specific association (17, 26–28). Reaction mixtures and amplification conditions were performed under standard conditions (21). Thus, the protocol used to perform the amplifications included a total volume of 50 μL containing 2U Taq Polymerase (Invitrogen®, ON, Canada), 2X Taq buffer (5 mM KCl Tris-HCl, pH 8.5), 1.5 mM MgCl2, 0.1 mM dNTPs (Promega®, Madison, WI, USA), and 5.0 mM of each primer (Promega®, Madison, WI, USA), and 2 μL of DNA. The amplification parameters included a first incubation for 5 min at 94°C, followed by 40 amplification cycles that consisted of a 30 s denaturing step at at 60°C (reactions 1 and 2) or 65°C (reaction 3), and a 1 min elongation step at 72°C, and a final extension step of 5 min. The gels were stained with Gel Red (Invitrogen®, Carlsbad, CA, USA), the amplicons were resolved by 5% agarose gel electrophoresis at 120 V for 1 h, and band visualization was carried out with a UV-transilluminator (Vilber Lourmat, Collegien, France). The concentration of agarose used was suitable for the expected band sizes. The stained gel was captured on a desktop computer using the Infinity® software (Tallahassee, FL, USA). Two control strains were used, *S*. Typhimurium 14,028 strain and *S*. Typhi STH 2,370 strain. Nuclease-free water was used as a negative control. For each serotype, the results of the multiplex PCR were analyzed through the construction of a binary matrix using “1” for presence and “0” for absence of amplified bands from each isolate. This matrix was used to calculate the similarity of the isolates by a hierarchical clustering, using the average linkage method and Jaccard's distance, and was represented by a dendrogram. This analysis was carried out using the Infostat software (29).

To determine the diversity of genotypes for each bacterial serotype, the Gini coefficient was calculated, which values range between “0” and “1”. In this coefficient, “0” suggests an equal distribution of isolates across all genotypes, and “1” represents one genotype as the unique source for all isolates (30).

### Serotyping

Once bacteria were genotyped by the multiplex PCR, one isolate representative of each genotype was sent to the national reference laboratory Instituto de Salud Pública de Chile (ISP), where they were serotyped following the Kauffman-White scheme (18).

### Detection of virulence genes

All isolated *S. enterica* strains were subjected to PCR for detection of seven virulence genes, including *spvC, pagK, sirA, gipA, prot6e, SEN1417* and *pefA* (31–33). The PCR reactions were performed with primers described in Supplementary Table S2, in a total volume of 25 μL containing 1U Taq Polymerase (Invitrogen®, ON, Canada), 1X Taq buffer (5 mM KCl Tris-HCl, pH 8.5), 1.5 mM MgCl2, 0.1 mM dNTPs (Promega®, Madison, WI, USA), 1 μM of forward and reverse primers (Promega®, Madison, WI, USA), and 1 μL of DNA. An incubation at 95°C for 10 min was used as an initial denaturation step followed by 35 cycles of amplification. Each cycle consisted of a denaturation step at 95°C for 1 min, followed by 1 min of annealing at different temperatures according to the target gene and elongation at 72°C for 1 min. The final elongation step was conducted at 72°C for 10 min. Previously sequenced strains harboring the target genes were used as controls (9). Supplementary Table S2 describes the annealing temperatures of each virulence gene and the GenBank accession number. A *Salmonella* strain that was negative for all virulence genes was used as a negative control. The amplicons were resolved by agarose gel electrophoresis (1.5 or 2.0% agarose) at 120 V for 1 h, and band visualization was carried out with Gel Red stain (Invitrogen®, Carlsbad, CA, USA) and a UV-transilluminator (Vilber Lourmat, Collegien, France) using the Infinity® software (Tallahassee, FL, USA).

### Phenotypic antimicrobial susceptibility testing

All isolated *S. enterica* strains were analyzed by the Kirby Bauer technique, according to the recommendations of the Clinical and Laboratory Standards Institute (34). The *Escherichia coli* ATCC 25,922 and *Klebsiella pneumoniae* ATCC 700,603 strains were used as quality controls. The bacterial inoculum was standardized to 0.5 McFarland units using a nephelometer. The antibiotics analyzed included ampicillin (AMP, 10 μg), amoxicillin + clavulanic acid (AMC, 20/10 μg), cefadroxil (CFR, 30 μg), ceftazidime (CAZ, 30 μg), ceftiofur (EFT, 30 μg), ceftriaxone (CRO, 30 μg), ciprofloxacin (CIP, 5 μg), gentamicin (CN, 10 μg), nalidixic acid (NA, 30 μg), sulfamethoxazole + trimethoprim (SXT, 23.75/1.25 μg), tetracycline (TE, 30 μg), streptomycin (S, 10 μg), azithromycin (AZM, 15 μg), enrofloxacin (ENR, 5 μg), trimethoprim (W, 5 μg), sulfisoxazole (SF, 300 μg), chloramphenicol (C, 30 μg), and fosfomycin (FOS, 200 μg), using commercial disks (Oxoid®, UK). Multidrug resistance (MDR) was defined as the resistance to three or more antimicrobial classes (35). Strains were analyzed for critically important antimicrobials, as defined by the World Health Organization (WHO) (36). For enrofloxacin, ciprofloxacin breakpoints were used; and the values for azithromycin were analyzed based on Parry et al. (37) and Martínez-Cortés et al. (38). The resistant isolates with an inhibition diameter less than or equal to the breakpoints for cefotaxime, ceftazidime, or ceftiofur were also examined to identify extended spectrum β-lactamses (ESBL) production, using the phenotypic confirmatory test with a cefotaxime + clavulanic acid disk (30/10 μg) or a ceftazidime + clavulanic acid disk (30/10 μg) (34). Intermediate strains were classified as resistant (39). The multiple antimicrobial resistance (MAR) index was calculated as “a/b”, where “a” corresponds to the number of antimicrobials for a particular isolate was resistant and “b” the total number of antimicrobials tested (40).

### Statistical analysis

Categorical data analyses were made through contingency tables with Infostat (2010v) software (29).

## Results

### Isolation and serotyping of *S*. *enterica*

From the 500 fecal samples collected from pigs, *S. enterica* strains were isolated in 9.2% of the sampled animals (*n* = 46). Among those strains, most corresponded to *S*. Typhimurium (60.9%, *n* = 28), followed by *S*. Infantis and *S*. Derby (15.2% each, *n* = 7), *S. enterica* group B (6.5%, *n* = 3), and *S. enterica* subsp. *enterica* rough strain (2.2%, *n* = 1). On the other hand, *S. enterica* strains were isolated in 19% (*n* = 57) from the 300 samples obtained from chickens, which corresponded to *S*. Infantis (56.1%, *n* = 32), *S*. Typhimurium (22.8%, *n* = 13), and *S*. Enteritidis (21.1%, *n* = 12) (Table 1).

**Table 1:** Number of isolates, genotypes and Gini coefficients of serotypes detected in pigs (P) and chickens (C).

**Serotype**	**N°isolates** **(P/C)**	**N°genotypes** **(P/C)**	**Gini** **(P/C)**
*S*. Typhimurium	41 (28/13)	18 (10/8)	0.4 (0.393/0.317)
*S*. Infantis	39 (7/32)	19 (5/14)	0.37 (0.171/0.388)
*S*. Enteritidis	12 (0/12)	9 (0/9)	– (–/0.204)
*S*. Derby	7 (7/0)	2 (2/0)	– (0.21/–)
*S*. Group B	3 (3/0)	3 (3/0)	– (0.0/–)
*S. enterica* subsp. *enterica* rough strain	1 (1/0)	1 (1/0)	– (0.0/–)

P, pigs; C, chickens; –, not calculated.

### Typing of *S*. *enterica* isolates using the multiplex PCR

The 46 isolates from pigs were typed by PCR and grouped into 21 different genotypes (Table 1 and Supplementary Table S3), and the 57 isolates from chickens were grouped into 31 different genotypes (Table 1 and Supplementary Table S4). Each genotype was always associated to only one serotype, although each serotype grouped several genotypes.

**Figure 1:**
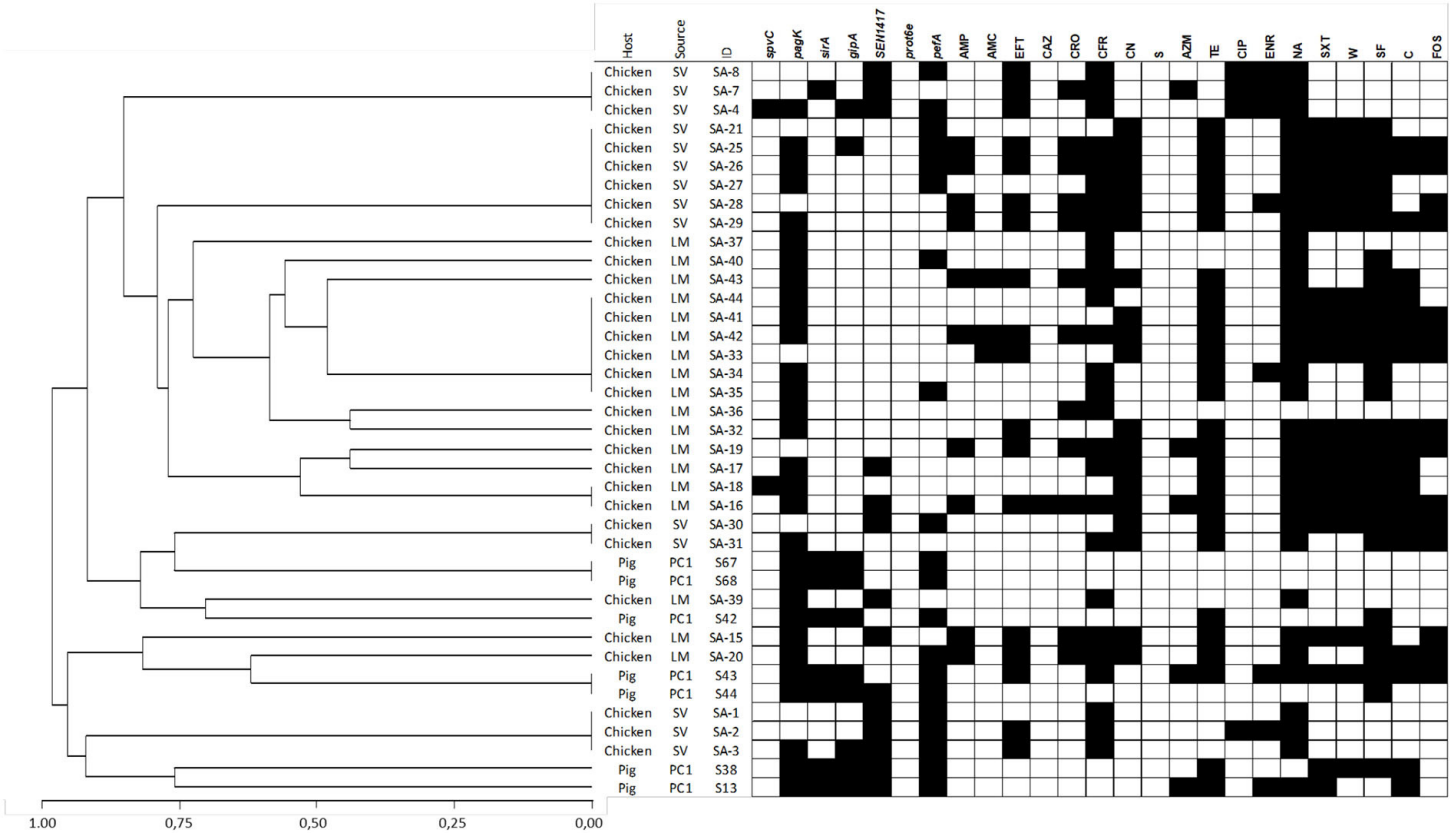
Dendrogram showing genetic similarities (%) among *S*. Infantis isolates analyzed using multiplex PCR. Detection of virulence-associated genes and antimicrobial resistance phenotypes are depicted as black squares when present. The dendrogram was constructed using a hierarchical clustering, using the average linkage method and Jaccard's distance.

**Figure 2:**
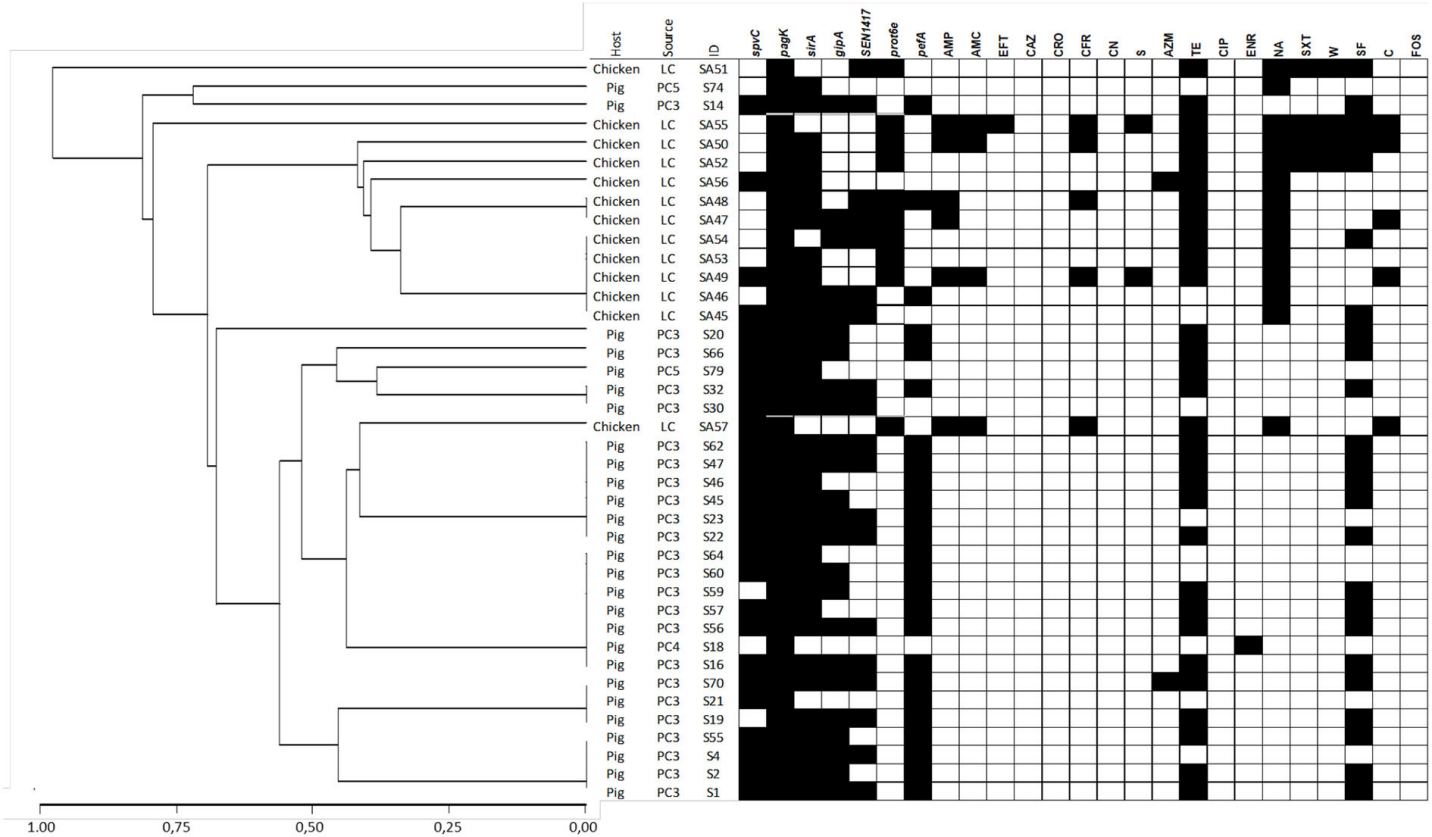
Dendrogram showing genetic similarities (%) among *S*. Typhimurium isolates analyzed using multiplex PCR. Detection of virulence-associated genes and antimicrobial resistance phenotypes are depicted as black squares when present. The dendrogram was constructed using a hierarchical clustering, using the average linkage method and Jaccard's distance.

The Gini coefficient varied according to the *Salmonella* serotype and animal species, ranging from 0.171 to 3.93 (Table 1). When this coefficient is calculated with all isolates by serotype, *S*. Infantis showed a slightly higher diversity than *S*. Typhimurium (Gini = 0.37 and 0.4, respectively), grouping 19 and 18 genotypes, respectively (Table 1). Most of them were represented by one or two isolates (Figures 1, 2 and Supplementary Tables S3, S4).

### Phenotypic antimicrobial resistance characterization of *S. enterica* strains

**Figure 3:**
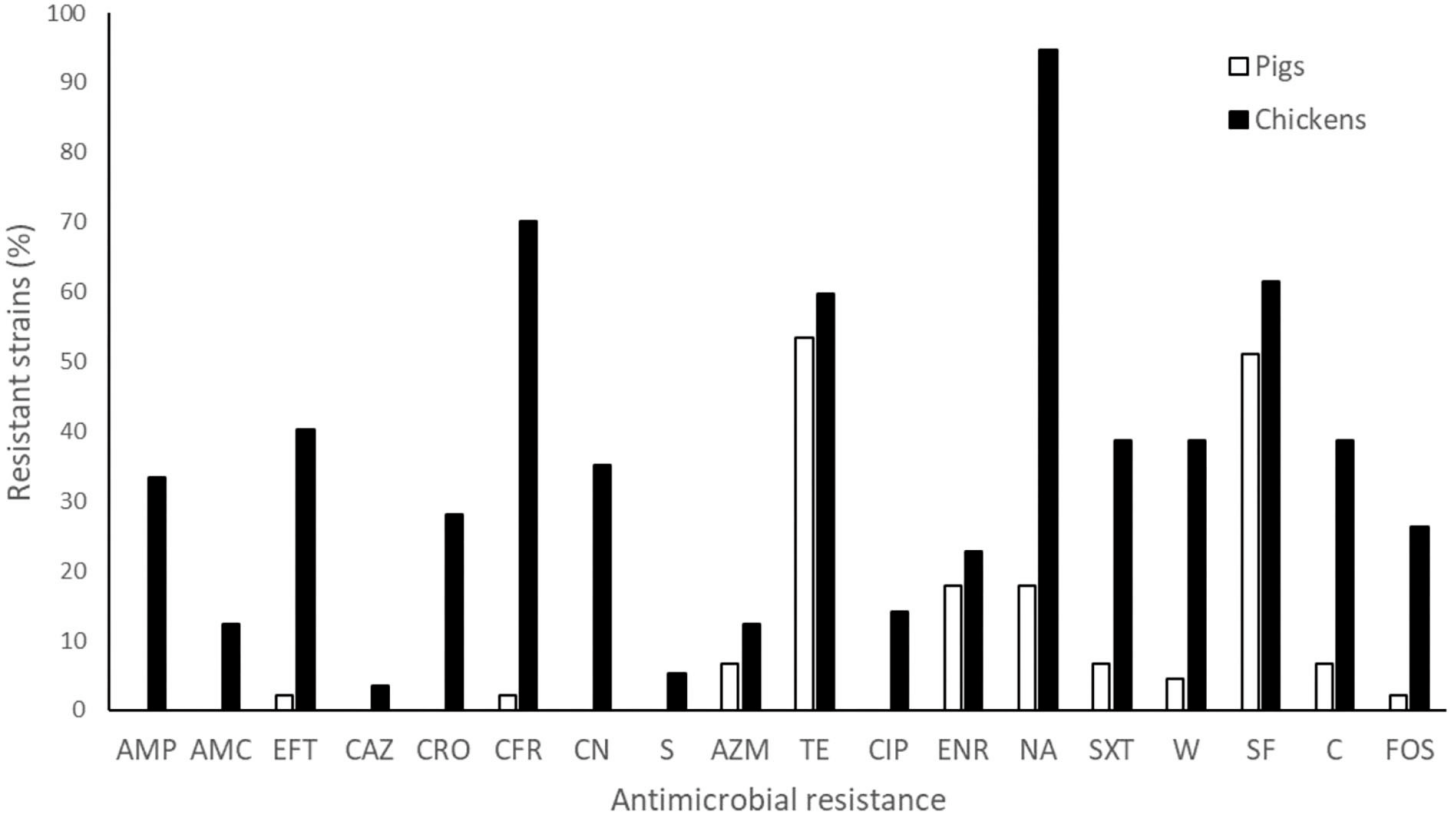
Phenotypic antimicrobial resistance of *S. enterica* strains isolated from pigs and chickens against 18 classes of antibiotics. AMP, ampicillin; AMC, amoxicillin + clavulanic acid; CFR, cefadroxil; CAZ, ceftazidime; EFT, ceftiofur; CRO, ceftriaxone; CIP, ciprofloxacin; CN, gentamicin; NA, nalidixic acid; SXT, sulfamethoxazole + trimethoprim; TE, tetracycline; S, streptomycin; AZM, azithromycin; ENR, enrofloxacin; W, trimethoprim; SF, sulfisoxazole; C, chloramphenicol; FOS, fosfomycin.

Most of the *S. enterica* strains isolated from pigs were resistant to at least one antibiotic (73.3%), with resistances against tetracycline (53.3%) and sulfisoxazole (51.1%) being the most frequent. Despite 10 phenotypic resistance profiles were detected, only 4 isolates (8.9%) showed MDR (Supplementary Table S5). In contrast, 98.2% of the *S*. *enterica* strains isolated from chickens were resistant to at least one drug and 82.5% presented MDR. This high AMR level was mainly observed with nalidixic acid (94.7%), cefadroxil (70.2%) and sulfisoxazole (61.4%) (Figure 3 and Supplementary Table S6), and determined 46 phenotypic resistance profiles (Supplementary Table S6). Within the *Salmonella* strains resistant to cefotaxime, ceftazidime and ceftiofur isolated from chicken, 32% were ESBL positive; while all strains isolated from pigs were ESBL negative. Furthermore, the highest MAR index value (0.8, a = 14, b = 18) was found in a *Salmonella* strain isolated from chicken.

### Distribution of virulence genes among *S. enterica* strains

The most prevalent virulence genes among the pig isolates were *pagK* and *sirA* (95.6% each), *pefA* (80%), *gipA* (62.2%), *spvC* (53.3%), and *SEN1417* (37.8%). Twelve virulotypes were detected, with *spvC*-*pagK*-*sirA*-*gipA*-*SEN1417*-*pefA* as the most frequent (26.7%) (Supplementary Table S7). No isolates harbored the *prot6e* gene.

In the case of *S. enterica* strains isolated from chickens, the genes *pagK* (68.4%), *SEN1417* (42.1%), *pefA* (28.1%), *sirA* (26.3%), *prot6e* (17.5%), *gipA* (14%), and *spvC* (12.3%) were detected. In these strains, 25 virulotypes were registered, with *pagK* alone as more frequently detected (21.1%) (Supplementary Table S8).

## Discussion

Understanding the epidemiology of *S. enterica* in different animal reservoirs is essential to achieve sanitary control of this pathogen and reduce its transmission to humans. Our results show several differences between strains isolated from two food-producing animal species, such as pigs and chickens. First, the rate of *S. enterica* isolation in feces of pigs was 9% and 19% in chickens. At national level, the isolation rate from pigs is higher than that reported previously by San Martín et al. (41), but lower than that reported by Lapierre et al. (42), with rates of 4.5 and 11.9% respectively. In the case of isolation rates from poultry, and as far as we know, to date there are no published studies. On the other hand, and at international level, our isolation rates differ from those of other authors. In this context, Ishihara et al. (43) reported a prevalence of *Salmonella* spp. of 2.9% and 36.1% in pigs and broiler chickens, respectively. More recently, Chen et al. (44) in China registered isolation rates of 44 and 9% in pork and chickens, respectively. These differences in detection rates may be due to variations in biosecurity measures for controlling bacterial contamination during the productive cycle of animals and processing of their carcasses. Moreover, the increased use of low-risk and certified feed ingredients in Chile could also contribute to lower residual contamination in animals, facilitating the traceability for detection of other sources of infection. However, differences between studies can also be attributed to specific sample sizes and isolation methods, the local environmental conditions and husbandry practices, among other factors. Regarding methodologies for the isolation of *Salmonella* from animal feces, the ability of the laboratory method to recover *Salmonella* from a sample containing a low concentration of this bacterium can be affected by biological factors such as competing microbiota and technical factors such as the culture media used, the volume of the feces and the pre-enrichment of the samples. We performed the isolation based on that described by Jensen et al. (24). These authors propose to add novobiocin to the BPW in the pre-enrichment stage. The authors point out that this method is more sensitive and that it increases the growth of *Salmonella* in the samples, although they also point out that 13 positive samples were lost. The conclusion of the authors is that the increased growth of *Salmonella* in novobiocin medium is due to a reduction of competitive microorganisms. So, it is possible that with the addition of novobiocin to BPW, we were not able to isolate *Salmonella* from all contaminated samples, and probably the actual positivity rate in our samples was higher. However, it is important to note that the animals sampled were healthy animals and therefore could excrete a low concentration of *Salmonella*, which might not be isolated from a sample with competitive microbiota (45). Thus, there are advantages and disadvantages to the addition of novobiocin in BPW for *Salmonella* isolation that should be evaluated by laboratories according to their objectives.

On the other hand, the most frequent serotype found in pig farms was *S*. Typhimurium, while in chicken farms was *S*. Infantis, in agreement with other reports from United States and the European Union (46–50). In particular, *S*. Infantis is considered an emerging serotype in the poultry industry across the world, associated with the acquisition of a pESI-like megaplasmid conferring MDR phenotypes (9, 51–54). Furthermore, both *S*. Infantis and *S*. Typhimurium have been ranked among the top 10 most frequently *Salmonella* serotypes involved in human infections worldwide, representing a food safety and public health hazard (55). In Chile, these serotypes occupy the 2^nd^ and 3^rd^ position in frequency, respectively, after *S*. Enteritidis (56); representing in aggregate around 75% of all clinical cases during last years.

The agglutination techniques for *Salmonella* serotyping does not have the required discriminative power to describe transmission chains during outbreaks investigations. Therefore, other quick, cost-effective resolution procedures have been studied, such as the multiplex PCR technique. In this work, this assay was performed for genotyping *Salmonella* isolates, detecting 21 different genotypic patterns in 46 strains isolated from pigs, and 31 distinct genotypic patterns in 57 strains from poultry. This means that genomic polymorphisms harbored by circulating *S. enterica* serotypes are giving rise to a high genotypic diversity of *Salmonella* isolates in both hosts, with Gini coefficients lower than 0.4 in all of them (Table 1). Apparently, there are few *Salmonella* serotypes very well adapted for survival into industrial environments, which infect animals after having been transmitted from various sources. In this context, industry-adapted *S. enterica* strains show high biofilm production, abiotic stress adaptation and extracellular adhesion; as well as adaptation to their poultry hosts (57–61). This scenario contrasts with the previous results of Kim et al. (17), Beaubrun et al. (20), and Beaubrun et al. (21), which suggested a unique genotype-serotype relationship. The genetic variability observed in our strains might be associated with several contributing factors, such as the origin of the animals and their food (62), the contamination in the premises by direct and/or indirect contact with humans (62), domestic animals or wild birds (63), or the presence of other carriers acting as vehicles of *S. enterica* strains (64, 65). For this reason, the inclusion of routine testing and categorization of animal herds on its *S. enterica* infection status are important components of integrated programs focused at reducing its prevalence in the food chain and the risks for animal and public health. Additionally, the observed diversity establishes the need of future analyses to determine the capability of multiplex PCR for supporting studies like that, and its potentiality for complementing the traditional serotyping through its discriminatory ability at a sub-serotype level. In this regard, it is important to highlight that the same genotype was not shared between serotypes, maintaining the specificity of the method. Furthermore, this molecular typing method suggests that pig and chicken industries are not sharing isolates (Figures 1, 2), being affected by independent infection sources which would require specific efforts to characterize *Salmonella* transmission chains and the implementation of their control methods.

A variety of virulence factors have been shown to play different roles in the pathogenesis of *Salmonella* infections. To perform virulotyping in *Salmonella* it is important to choose genes that are not present in 100% of the strains, based on the literature, in order to determine the variability of the isolated strains. Regarding the detection of virulence genes, we found 25 different virulotypes in chicken and 12 different virulotypes in pig. The most detected genes were *pagK* (68%) and *SEN1417* (42.1%). In fact, the *pagK* gene, as the only amplified gene, was the most frequent virulotype (*n* = 12; 21%), corresponding to serotypes *S*. Infantis (*n* = 10) and *S*. Enteritidis (*n* = 2). The high frequency of *pagK* in pigs (95.6%) and chicken (68.4%) suggests its association with an important function during bacterial colonization of animal hosts, codifying a translocated factor into the cytosol of host cells (66), which also participates in biofilm formation (9).

A striking result is the difference in the number of antibiotic resistant strains in both food animal industries. The strains isolated from pigs showed low AMR levels and no critical drug resistances were detected. On the contrary, *S. enterica* strains isolated from poultry were much more resistant, including resistance against critical antibiotics, especially to cephalosporins. In Chile, the commercial pig and chicken farms have developed a high technological, biosecurity and sanitary related standards. They are intensive, vertically integrated, with restrictions on the use of antibiotics and homogeneity on their sanitary and productive management. Then, the contrasting findings in the AMR profiles could suggest a serotype effect, especially associated to *S*. Infantis, or a management effect, related to differences in the antibiotic usage in both type of productions. In this regard, emerging strains of *S*. Infantis carrying the megaplasmid pESI show a MDR phenotype; and have been described in several countries, including Chile (67–69). It is therefore very likely that the *S*. Infantis strains, mostly isolated from chickens, contains this megaplasmid and, as a consequence, express a MDR phenotype and develop a higher virulence than non-MDR strains (70). In the case of pigs, four strains were MDR, one of which showed simultaneous resistance against 10 antibiotics. In the case of *S. enterica* strains isolated from chickens, 33 strains presented a MDR phenotype, with two of them resistant to 10 antibiotics, two to 11 antibiotics, and even two to 12 antibiotics. In this study, a high MAR rate (>0.50) was observed in 9 (15.8%) strains isolated from chickens, indicating that *Salmonella* strains isolated from chickens express higher levels of multi-resistance compared to *Salmonella* strains isolated from pigs.

In Chile, despite regulations for the use of antibiotics in food-producing animals have been implemented (71), the most common antimicrobials used in pig or broiler chicken production are unknown. Moreover, ceftiofur, which is a third-generation cephalosporin, is permitted in these species. Consequently, bacteria isolated from chickens expressed resistance against critical antibiotics indicated by the WHO and OIE, such as cephalosporins, especially ceftiofur (39%), and fosfomycin (33%) (36, 72). In this context, Lai et al. (73) in China, also described high (42%) ceftiofur resistance rates in *Salmonella* strains isolated from chickens, pigs and ducks. The importance of monitoring the resistance to ceftiofur relies on its similar mechanism of action with ceftriaxone, a cephalosporin often used to treat *Salmonella* infections in children (74). Therefore, the increased rate of ceftiofur resistant *Salmonella* strains of animal origin may has important public health implications, limiting therapeutic alternatives in human medicine. In South America, there is limited information about AMR associated to food-producing animals. Among the scarce information available, Vinueza-Burgos et al. (75) in Ecuador reported AMR to critical drugs, including 5.8% to ceftazidime, 98% to nalidixic acid, and 94% to ciprofloxacin. In our study, among the *Salmonella* strains isolated from chickens, we found 15% of enrofloxacin resistant strains, and similarly to Vinueza-Burgos et al. (75), our isolates were also highly resistant to nalidix acid (80%). The high percentage of resistance to this antibiotic could be reflected in the future with higher AMR to fluoroquinolones, such as enrofloxacin, a risk that deserves a close surveillance. Additionally, 33% of isolates from chickens were resistant to fosfomycin, which is a recently reintroduced antibiotic for the treatment of acute urinary tract infections in humans (76). Several studies in *Enterobacteriales* have reported the simultaneous presence of fosfomycin resistance genes with β-lactamases encoding genes (77, 78). In this work 17.5% of isolates showed both phenotypes of resistance, a situation that can also be considered of concern for public health.

In summary, this study suggests that in pig farms located in central Chile the predominant *S. enterica* serotype is *S*. Typhimurium and in chicken farms is *S*. Infantis, with a genotypic diversity implying different sources and transmission chains. Isolates from poultry showed more resistance phenotypes against antibiotics, with critical importance those associated with cephalosporins, fosfomycin and fluoroquinolones. The presence of genes, related with virulence and phenotypic antibiotic resistance, indicate a convergence of virulence and resistance determinants, highlighting that efforts should be focused on implementing strict farm-to-table surveillance programs for this pathogen in industrial pig and poultry farms, in order to prevent outbreaks in the population.

## Data availability statement

The original contributions presented in the study are included in the article/Supplementary material, further inquiries can be directed to the corresponding author/s.

## Ethics statement

Ethical review and approval was not required for the animal study because Study was performed with resistant bacteria. Samples where taken by authorized veterinarians working in the farms. Researchers do not perform any animal studies.

## Author contributions

PR and LL contributed to the conception, design of the study, and wrote the first draft of the manuscript. PR, LL, LS, and JC contributed with resources to the study. JG, MB, and TA performed the laboratory analyses. PR, LL, and JC performed the statistical analysis. LL, NG, LS, and JC wrote sections of the manuscript. All authors contributed to the article and approved the submitted version.

## Funding

This work was self-founded by JC and LL.

## Conflict of interest

The authors declare that the research was conducted in the absence of any commercial or financial relationships that could be construed as a potential conflict of interest.

## Publisher's note

All claims expressed in this article are solely those of the authors and do not necessarily represent those of their affiliated organizations, or those of the publisher, the editors and the reviewers. Any product that may be evaluated in this article, or claim that may be made by its manufacturer, is not guaranteed or endorsed by the publisher.
