# Supplementary material for: Virulence and antimicrobial resistance factors in Salmonella enterica serotypes isolated from pigs and chickens in central Chile
Source: Front Vet Sci. 2022 Sep 20;9:971246. doi: 10.3389/fvets.2022.971246 (PMC9530323; doi:10.3389/fvets.2022.971246)
Supplement: Supplementary file 1 [file Data_Sheet_1.pdf]

Table S1. Primer sequences and bands ID for the molecular typing of *S. enterica*.

Reaction	Primer name	Primers sequence (5´-3´)	Product size (bp)	Band ID	Access numbers
Reaction 1	STM0716F<br>STM0716R	AACCGCTGCTTAATCCTGATGG<br>R: TGGCCCTGAGCCAGCTTTT	187	A	CP098834.1
	STM1350F<br>STM1350R	TCAAAATTACCGGGCGCA<br>TTTTAAGACTACATACGCGCATGAA	171	B	CP098834.1
	STM0839F<br>STM0839R	TCCAGTATGAAACAGGCAACGTGT<br>GCGACGCATTGTTCGATTGAT	137	C	CP098741.1
	STM4525F<br>STM4525R	TGGCGGCAGAAGCGATG<br>CTTCATTCAGCAACTGACGCTGAG	114	D	CP098741.1
	STM4538F<br>STM4538R	TGGTCACCGCGCGTGAT<br>CGAACGCCAGGTTCATTTGT	93	E	CP098741.1
Reaction 2	STY0311<br>STY0312	TGGTATGGTTAAGCGGAGAATGG<br>GAGAGTCATAGCCCACACCAAAG	301	F	CP093132.1
	STY0346<br>STY0347	GGCTGGAGCAGCCTTACAAAA<br>AAGAGTTGCCTGGCTGGTAAAA	262	G	CP098831.1
	STY2299<br>STY2300	AATCCCCCCCCCTCAAAAA<br>GGTACACGTTTACTGTTTGCTGGA	220	H	CP097262.1
	STM3845F<br>STM3845R	ATATCTCATCGTCTCCTTTTCGTGT<br>GAAGGTCCGGATAGGCATTCT	181	I	CP098741.1
	STY2349F<br>STY2349R	AATTACGGAGCAGCAGATCGAGG<br>TGCGGCCAGCTGTTCAAAA	124	J	CP096171.1
Reaction 3	PT4F<br>PT4R	GGCGATATATAAGTACGACCATCATGG<br>GCACGCGGCACAGTTAAAA	225	K	CP097262.1
	STM2150F<br>STM2150R	CATAACCCGCCTCGACCTCAT<br>AGATGTCGTGAGAAGCGGTGG	101	L	CP098741.1

Table S2. Primer sequences for detection of virulence-associated genes.

Gene	Primers sequence (5´-3´)	Product size (bp)	Annealing T°	Access numbers	Reference
pefA	F: CCTGTGACCTGACCACTTCTG<br>R: GTAAGCCACTGCGAAAGATG	418	51°C	CP088139.1	(1)
spvC	F: CTCCTTGCACAACCAAATGCG<br>R: TGTCTCTGCATTTCACCACCATC	570	53°C	CP088135.1	(1)
sirA	F: TGCGCCTGGTGACAAAACTG<br>R: ACTGACTTCCCAGGCTACAGCA	313	55°C	CP098831.1	(1)
gipA	F: ACGACTGAGCAGGCTGAG<br>R: TTGGAAATGGTGACGGTAGAC	518	58°C	CP098741.1	(1)
SEN1417	F: GATCGCTGGCTGGTC<br>R: CTGACCGTAATGGCGA	670	58°C	CP050716.1	(2)
pagK	F: ACCATCTTCACTATATTCTGCTC<br>R: ACCTCTACACATTTTAAACCAATC	151	60°C	CP098741.1	(3)
prot6e	F: GCCTAAGGTTAGTGTGACTCTC<br>R: CTAGCAGCCGTTGGTATCC	579	50°C	CP092322.1	(1)

1. Huehn S, La Ragione RM, Anjum M, Saunders M, Woodward MJ, Bunge C, et al. Virulotyping and Antimicrobial Resistance Typing of Salmonella enterica Serovars Relevant to Human Health in Europe. Foodborne Pathogens and Disease. 2010 May;7(5):523–35.

2. Pan Z, Carter B, Núñez-García J, AbuOun M, Fookes M, Ivens A, et al. Identification of genetic and phenotypic differences associated with prevalent and non-prevalent Salmonella Enteritidis phage types: analysis of variation in amino acid transport. Microbiology. 155(10):3200–13.

3. Huehn S, Bunge C, Junker E, Helmuth R, Malorny B. Poultry-Associated Salmonella enterica subsp. enterica Serovar 4,12:d:− Reveals High Clonality and a Distinct Pathogenicity Gene Repertoire. Applied and Environmental Microbiology. 2009 Feb 15;75(4):1011–20.

Table S3. Serotypes and genotypes of *S. enterica* strains isolated from pigs.

Serotype (N° of isolates)	Source (farm)	Genotypic banding pattern	Genotype	Number of isolates (%)
S. Infantis (7)	PC1	ABGL	G1	2 (4.3)
	PC1	ABF	G2	2 (4.3)
	PC1	ABHI	G8	1 (2.2)
	PC1	BI	G9	1 (2.2)
	PC1	BCDGL	G12	1 (2.2)
S. Derby (7)	PC4	ABCJL	G3	2 (4.3)
	PC4	ABCEJL	G5	5 (10.9)
S. Group B (3)	PC4	ABCEJ	G6	1 (2.2)
	PC4	ACJ	G7	1 (2.2)
	PC3	ABCDHIL	G15	1(2.2)
S. Typhimurium (28)	PC5	BCEJL	G4	1 (2.2)
	PC3	CDJL	G10	1 (2.2)
	PC3	ACDHIJ	G13	1 (2.2)
	PC3	BCHIL	G14	2 (4.3)
	PC5	ABCDHIL	G15	1 (2.2)
	PC3	BCDHIL	G16	2 (4.3)
	PC3	ACDHIL	G17	6 (13.0)
	PC3-PC4	CDHIL	G18	7 (15.2)
	PC3	BCDIL	G19	3 (6.5)
	PC3	CDIL	G20	4 (8.7)
S. enterica subsp. *enterica* rough strain (1)	PC1	BCDGIL	G11	1 (2.2)

Table S4. Serotypes and genotypes of *S. enterica* strains isolated from chicken.

Serotype (N° of isolates)	Source (Farm)	Genotypic banding pattern	Genotype	Number of isolates (%)
S. Enteritidis (12)	SV	ACDGJ	G44	2 (3.5)
	SV	BCDHJ	G45	1 (1.8)
	SV	BCEFJL	G46	1 (1.8)
	SV	BCEGJ	G47	1 (1.8)
	SV	BCG	G48	1 (1.8)
	LM	BCGH	G49	1 (1.8)
	SV	BCI	G50	1 (1.8)
	LM	BFGK	G51	1 (1.8)
	SV	C	G52	3 (5.2)
S. Infantis (32)	LM	BEF	G22	1 (1.8)
	LM	AEFGK	G23	2 (3.5)
	LM	AEFGKL	G24	1 (1.8)
	LM	AFGKL	G25	1 (1.8)
	SV(3)-LM(1)	AF	G26	4 (7.0)
	SV	AG	G27	6 (10.5)
	SV	BG	G28	5 (8.9)
	LM	ACEGK	G29	1 (1.8)
	LM	ACGK	G30	5 (8.8)
	LM	ACG	G31	2 (3.5)
	LM	ACEGKL	G32	1 (1.8)
	LM	ADGK	G33	1 (1.8)
	LM	BDEGKL	G34	1 (1.8)
	LM	ACGHK	G35	1 (1.8)
S. Typhimurium (13)	LC	ABCDFIKL	G36	5 (8.8)
	LC	ABCDFHIKL	G37	2 (3.5)
	LC	ACDFIKL	G38	1 (1.8)
	LC	ABCDIKL	G39	1 (1.8)
	LC	AIL	G40	1 (1.8)
	LC	ABDFIKL	G41	1 (1.8)
	LC	ACDIL	G42	1 (1.8)
	LC	BEFG	G43	1 (1.8)

Table S5. Phenotypic resistance profiles detected in *S. enterica* strains isolated from pigs.

Resistance profile	Number of strains (%)
TE-SF	19 (41.3)
AZM-TE-ENR-NA-SXT-C	1 (2.2)
ENR	2 (4.3)
ENR-NAL	4 (8.7)
TE-SXT-W-SF-C	1 (2.2)
EFT-CFR-AZM-TE-ENR-NA-SXT-W-SF-C-FOS	1 (2.2)
SF	1 (2.2)
AZM-TE-SF	1 (2.2)
NA	2 (4.3)
TE	1 (2.2)

AMP: ampicillin, AMC: amoxicillin + clavulanic acid, CFR: cefadroxil, CAZ: ceftazidime; EFT: ceftiofur: EFT, CRO: ceftriaxone, CIP: ciprofloxacin, CN: gentamicin, NA: nalidixic acid, SXT: sulfamethoxazole + trimethoprim, TE: tetracycline, S: streptomycin, AZM: azithromycin, ENR: enrofloxacin, W: trimethoprim, SF: sulfisoxazole, C: chloramphenicol, FOS: fosfomycin.

Table S6. Phenotypic resistance profiles detected in *S. enterica* strains isolated from chickens.

Resistance profile	Number of strains (%)
NA	2 (3.5)
CFR-NA	4 (7.0)
CRO-CFR	1 (1.8)
NA-SF	1 (1.8)
TE-NA	1 (1.8)
EFT-CFR-NA	1 (1.8)
ENR-NA-SF	1 (1.8)
CFR-ENR-NA	1 (1.8)
CFR-NA-SF	1 (1.8)
TE-NA-SF	1 (1.8)
AZM-TE-NA	1 (1.8)
EFT-CRO-CFR-SF	1 (1.8)
AMP-CFR-ENR-NA	1 (1.8)
CFR-TE-NA-SF	1 (1.8)
AMP-TE-NA-C	1 (1.8)
AMP-CFR-TE-NA	1 (1.8)
EFT-CFR-CIP-ENR-NA	3 (5.3)
CFR-TE-ENR-NA-SF	1 (1.8)
TE-NA-SXT-W-SF	2 (3.5)
EFT-CRO-CFR-CIP-ENR-NA	1 (1.8)
CN-TE-NA-SXT-W-SF	1 (1.8)
AMP-AMC-CFR-TE-NA-C	1 (1.8)
AMP-CFR-AZM-CIP-ENR-NA-SF	1 (1.8)
EFT-CRO-CFR-AZM-CIP-ENR-NA	1 (1.8)
CN-TE-NA-SXT-W-SF-C	1 (1.8)
EFT-CFR-AZM-CIP-ENR-NA-SF	1 (1.8)
CFR-CN-TE-NA-SXT-W-SF	1 (1.8)
CFR-CN-TE-NA-SF-C-FOS	1 (1.8)
CFR-TE-NA-SXT-W-SF-C	1 (1.8)
AMP-AMC-CFR-S-TE-NA-C	1 (1.8)
CFR-CN-TE-NA-SXT-W-SF-C	1 (1.8)
CN-TE-NA-SXT-W-SF-C-FOS	2 (3.5)
EFT-CRO-CFR-S-AZM-CIP-ENR-NA-SF	1 (1.8)
EFT-CN-TE-NA-SXT-W-S-C-FOS	1 (1.8)
AMP-AMC-CFR-TE-NA-SXT-W-SF-C	1 (1.8)
AMP-EFT-CRO-CFR-CN-TE-NA-SF-C-FOS	1 (1.8)
AMC-EFT-CN-TE-NA-SXT-W-SF-C-FOS	1 (1.8)
AMP-AMC-EFT-CRO-CFR-CN-TE-NA-SF-C	1 (1.8)
AMP-EFT-CRO-CFR-CN-TE-NA-SXT-W-SF-FOS	1 (1.8)
AMP-AMC-EFT-CFR-S-TE-NA-SXT-W-SF-C	1 (1.8)
AMP-EFT-CRO-CFR-CN-TE-NA-SXT-W-SF-C-FOS	3 (5.3)
AMP-EFT-CRO-CFR-CN-TE-ENR-NA-SXT-W-SF-FOS	1 (1.8)
AMP-EFT-CAZ-CRO-CFR-CN-TE-NA-SXT-W-SF-C-FOS	1 (1.8)
AMP-EFT-CRO-CFR-CN-AZM-TE-NA-SXT-W-SF-C-FOS	1 (1.8)
AMP-AMC-EFT-CRO-CFR-CN-TE-NA-SXT-W-SF-C-FOS	1 (1.8)
AMP-EFT-CAZ-CRO-CFR-CN-AZM-TE-NA-SXT-W-SF-C-FOS	1 (1.8)

AMP: ampicillin, AMC: amoxicillin + clavulanic acid, CFR: cefadroxil, CAZ: ceftazidime; EFT: ceftiofur: EFT, CRO: ceftriaxone, CIP: ciprofloxacin, CN: gentamicin, NA: nalidixic acid, SXT: sulfamethoxazole + trimethoprim, TE: tetracycline, S: streptomycin, AZM: azithromycin, ENR: enrofloxacin, W: trimethoprim, SF: sulfisoxazole, C: chloramphenicol, FOS: fosfomycin.

Table S7. Virulotypes detected in *S. enterica* strains isolated from pigs.

Virulotype	Number of strains (%)
pagK	2 (4.3)
pagK-sirA	6 (13.0)
sirA-pefA	2 (4.3)
spvC-pagK-pefA	2 (4.3)
pagK-sirA-pefA	3 (6.5)
spvC-pagK-sirA	2 (4.3)
pagK-sirA-gipA-pefA	5 (10.9)
spvC-pagK-sirA-pefA	3 (6.5)
spvC-pagK-sirA-gipA-pefA	6 (13.0)
pagK-sirA-gipA-SEN1417-pefA	2 (4.3)
spvC-pagK-sirA-gipA-SEN1417	1 (2.2)
spvC-pagK-sirA-gipA-SEN1417-pefA	12 (26.1)

Table S8. Virulotypes detected in *S. enterica* strains isolated from chickens.

Virulotype	Number of strains (%)
SEN1417	5 (8.8)
pefA	2 (3.5)
sirA	2 (3.5)
pagK	12 (21.0)
SEN1417-pefA	4 (7.0)
sirA-prot6e	3 (5.2)
pagK-sirA	2 (3.5)
pagK-SEN1417	4 (7.0)
spvC-pagK	1 (1.8)
pagK-pefA	5 (8.8)
pagK-prot6e	1 (1.8)
pagK-gipA-pefA	1 (1.8)
pagK-sirA-prot6e	3 (5.2)
pagK-SEN1417-prot6e	1 (1.8)
spvC-pagK-sirA	1 (1.8)
spvC-pagK-prot6e	1 (1.8)
pagK-gipA-SEN1417-pefA	1 (1.8)
spvC-pagK-sirA-prot6e	1 (1.8)
pagK-gipA-SEN1417-prot6e	1 (1.8)
spvC-pagK-gipA-SEN1417-pefA	1 (1.8)
spvC-sirA-gipA-SEN1417-pefA	1 (1.8)
spvC-pagK-sirA-gipA-SEN1417	1 (1.8)
pagK-sirA-gipA-SEN1417-pefA	1 (1.8)
pagK-sirA-gipA-SEN1417-prot6e	1 (1.8)
pagK-sirA-SEN1417-prot6e-pefA	1 (1.8)

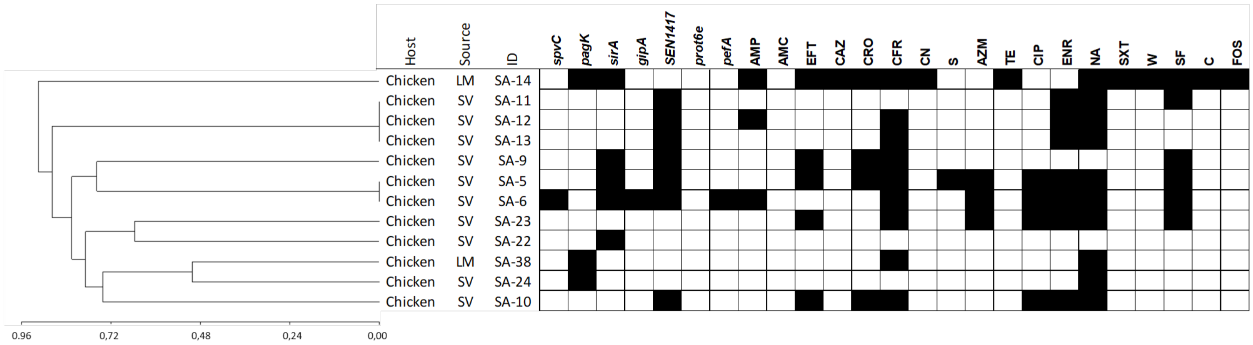

Figure S1. Dendrogram showing genetic similarities (%) between *Salmonella enterica* serotype Enteritidis isolates analyzed using multiplex PCR. Detection of virulence-associated genes and antimicrobial resistance phenotypes are depicted as black squares when present. The dendrogram was constructed using a hierarchical clustering, using the average linkage method and Jaccard's distance
